# Social Representation, Stereotypes and Social Identity Pertaining to Nursing Through Children's Drawings: A Qualitative Study

**DOI:** 10.1155/nrp/2417051

**Published:** 2024-12-31

**Authors:** E. Begoña García-Navarro, María José Cáceres Titos, Iván Rodríguez Pascual

**Affiliations:** ^1^Faculty of Nursing, University of Huelva, Avenida de las Fuerzas Armadas, s/n. 21007, Huelva, Spain; ^2^Center for Research in Social Studies and Social Intervention, Avenida Tres de Marzo s/n. 21071, Huelva 21071, Spain; ^3^Research Center for Contemporary Thought and Innovation for Social Development, Avenida de las Artes, s/n. 21007, Huelva, Spain; ^4^Faculty of Social Work, University of Huelva, Avenida de las Fuerzas Armadas, s/n.-21007, Huelva, Spain

**Keywords:** child, drawing, nursing education, qualitative research, social identity, social representation, stereotyping

## Abstract

**Aim:** The aim of this study is to explore children's depictions of nursing professionals, identifying shared and differential visual and symbolic elements as a function of gender, the period during which the drawing was made (pre-/postpandemic) and whether or not one has a relative who works in the same profession.

**Background:** Drawing circumnavigates the limits imposed by literacy and gives a voice to children who are able to express their personal feelings and subconscious through the drawn object. Theories of social representations and identity strive to explore the way in which children perceive and value the role of nurses in society.

**Methods:** With the question, ‘what is nursing to you?' providing the foundation, qualitative visual analysis was performed based on bottom-up inductive logic, analysing 105 drawings through the software CAQDAS Atlas-ti.

**Results:** Nine categories emerged that corresponded to uniform, typically used tools, represented functions, decorative elements, associated individuals, work setting and facial expression. Differences were identified as a function of the gender of the child respondent, familiarity with the profession and the period during which the drawing was made (pre-/postpandemic).

**Conclusion:** The present research strives to contribute towards understanding of perceptions of nursing figures in the symbolic world of children, taking a more complete look at the view children have of nursing and the way in which they conceive and value healthcare and caring for health.

**Summary**

• This study provides an in-depth look at the tasks and responsibilities that children associate with nurses, which can help to identify the aspects of the profession that are most attractive to children and the areas where education and public information about the profession need to be improved.
• The consolidated criteria for reporting qualitative research (COREQ) guidelines were used for reporting.
• The consumer researcher was involved in design, data analysis and publication preparation.

## 1. Introduction

Professional identity, formed through shared attitudes, values, knowledge and abilities among members of a community, evolves continuously throughout life, influenced by status, group dynamics and societal recognition [1]. Social image, defined as distinctive attributes, shapes collective professional identity, influenced by cultural factors, traditions, customs and ways of life that make a social and economic contribution to the identity of the group [2]. Despite the fact that the professional image of nurses has continuously evolved, according to a number of studies [3], stereotypes remain with regard to the profession, making it one of the most stigmatised professions historically [4]. The mass media has perpetuated an image that is far from the reality of nursing [5], erroneously associating nursing with the wearing of dresses, mob caps and heels. One published study [6] demonstrated that, to a certain extent, the professional identity of nurses comes from their public image and the pathway towards the formation of this professional identity begins prior to the onset of professional nursing studies.

Specifically, this first image is constructed from a very early age, following a first contact and experience with this professional practice. It is based on lived experiences, closeness to a nurse, the day-to-day actions of their professional role and the perception health centre visitors have of this [7]. Children's perceptions of nursing provide valuable insight into the way in which nursing identity is shaped and developed in society, in addition to the way in which these perceptions may impact attitudes and future understanding of children regarding nursing professionals.

## 2. Background

Nursing identity, which encapsulates aspects of nursing such as role, responsibilities and social image, can be analysed through the interpretation of children in different contexts such as drawings, symbolic games and interviews. According to social representation theory [8], representation is understood as discourse captured through an image, enabling communication regarding perceptions of things, individuals, feelings, moments and emotions. In this sense, reproducing the image of something, such as a drawing of a nurse, represents the apprehension of reality and socially constructed imaginary.

Thus, to talk about representation is to discuss communication about a part of this given social reality. In this sense, the traditional research paradigm must be left behind [9], transforming research so that it involves processes conducted with these realities instead of on them [10]. In this way, the application of creative and participative methodologies opens up the space and time to talk about diverse topics. This allows focus to be directed towards elements that are often relegated to taking a backseat, capturing aspects about a situation that would be impossible to record through oral or written observations alone [11]. The use of images or drawings enables one to express themselves and communicate freely, overcoming, in this way, the literacy barrier [12]. This enables a lot of information to be obtained in a short period of time and provides a clearly different and richer ‘new way of telling' [13].

A number of authors argue that drawing represents children's voices [14]. Indeed, according to many researchers, drawing is the main communication tool used by children before the age of six [15]. For infants, the conception or apprehension of an idea about something is loaded with meaning, which comes from their environment and their own experience with this something. For this reason, interpreting images created by children, on the one hand, brings us closer to their constructed reality and, on the other hand, concerns the way in which were construct the communication presented from the epistemological sphere.

Projective methods, such as drawing, are widely applicable to children and are often used in counselling, psychotherapy and clinical practice [16]. Projective methods have been revealed to be technically valid for use with children due to their nonthreatening nature and ability to open up the world inside children. Children's drawings can indicate their feelings and thoughts and reflect their nonverbal communication with the world. Children's drawings are viewed as explicit images of the internal models of various objects that help children express a visually realistic image of this object. Theories supporting drawing as a projective technique propose that personal and subconscious feelings towards an object are projected through images drawn by individuals. Subsequently, drawings may be used to assist in the evaluation of subconscious and sensitive aspects of children's behaviour.

In this sense, different international studies [17] have already used this technique in the area of paediatrics, gathering information on the voices and opinions of children. Specifically, a study conducted in Spain used children's drawings to analyse the need of children to play outside and relate with their peers during confinement [18].

In light of that presented above, there is a clear need to consider children's perceptions, placing greater emphasis on a social and inclusive education that promotes that the participation of children and public health. This is necessary given that current measures have led to low self-esteem, giving rise to negative emotions and a sense of abandonment in children and young people. Thus, different social spheres such as those linked with education, policy and health must make the effort to promote well-being in children and, for this, it is essential to ensure the active and real participation of children by making use of evaluation techniques and methodologies [19].

In consideration of the aforementioned, the present research seeks to explore children's depictions of nursing professionals, identifying shared and differential visual and symbolic elements as a function of gender, the period during which the drawing was made (pre-/postpandemic) and whether or not one has a relative who works in the same profession.

The pertinence of the present study is based on the need to understand potential differences in children's perceptions of the nursing figure before and after the pandemic through social representations. Analysis over time provides the opportunity to identify emerging patterns, comparing children's representations in both contexts, and enabling the possibility of identifying nuances that could be related with lived experiences during the pandemic.

Furthermore, this study explores potential sex differences in interpretations of the nursing figure. This gender perspective with regard to representations will enable a more nuanced understanding of the way in which these professional roles are perceived and conceptualised by both groups.

Likewise, potential disparities are considered between children as a function of whether or not they have relatives who work in the profession. This will enable a more in-depth look to be taken at the way in which personal experience and family context may influence the construction of children's perceptions with regard to nursing.

Finally, through analysis of children's perceptions of the nursing figure through their drawings, it will be possible to understand the way in which perceptions of the nursing figure have been integrated into their symbolic world. This will enable a more complete view of the influence of these professionals on the way in which children conceive and value healthcare and caring for health. Findings from the present study may also be useful for designing educational strategies that promote a more accurate and respectful image of nursing from an early age.

## 3. Methods

This study was conducted using a descriptive qualitative analysis based on social representations theory and identity regarding nursing practice.

Data collection started in September 2019 but had to be suspended in March 2020 due to the COVID-19 pandemic. The study was resumed in March 2021, with the final cases being added in November 2021.

The study was based on a visual analysis grounded in bottom-up or inductive coding logic. The aim of this was to enable progressive identification of the components emerging from examined drawings and the textual elements incorporated within them, until broader assessment categories were produced. Examined drawings were subjected to the fewest handling procedures required for scanning, incorporation, in their entirety, into analysis and, where necessary, anonymisation. Drawings were anonymised, and each participant was given a code number, assigned in order of completion (example: Drawing 1, Drawing 2, etc.). In accordance with the methodology employed by other similar studies [20], children's willingness to draw was taken as implicit consent. COREQ (COnsolidated criteria for REporting Qualitative research) were followed [21].

### 3.1. Participants

Drawings were gathered through second-year nursing degree students. The module titled Nursing Methodology contains content in line with the conceptualisation of nursing roles in society. Thus, directed academic activities were incorporated into this module to encourage placing students at the centre of their learning and, in this way, following the use of techniques such as drawing in children, a representation of the role of the nurse in current society was reached. Each nursing student selected a child from their close environment. Although we initially considered purposive sampling to ensure the heterogeneity of the informants creating the drawings, the students encountered significant accessibility challenges. Therefore, we decided to use a snowball sampling [22]. Prior to contacting the child, parents were contacted by students through an online questionnaire, which included an information sheet containing all information pertaining to the research project, alongside a consent form. Following the receipt of consent from parents, in accordance with pertinent guidelines, children were requested to produce a drawing, which was scanned and attached to the questionnaire.

Study participants were 105 children aged between four and 12 years coming from different public schools in Huelva province, in the South East of Spain.

Inclusion criteria required children to be aged between four and 12 years and undertaking schooling in any public school in the province of Huelva, Spain. Children who did not have the ability to draw or understand the meaning of a nurse from their perspective were excluded.

### 3.2. Procedure

This study was implemented in the following order. Children and their families were informed about the study. They were informed that participation was entirely voluntary, with no type of retribution, and that they were free to drop out of the study or withdraw their consent at any time. Following the receipt of parental written consent, drawings were gathered from children attending different schools throughout Huelva province. Parents delivered a 25 × 35 cm sheet of paper to children, who were instructed to ‘draw what a nurse is to you'. Following this, parents were requested to respond to sociodemographic questions, providing information about whether they themselves or close relatives of their children worked in the healthcare setting. Finally, they attached and sent a scanned copy of the drawing through Google Forms. Approximately 60 min was required to complete the sociodemographic questionnaire and the drawing task. The sample size was estimated to be sufficient based on the principle of theoretical saturation [23].

### 3.3. Data Analysis

In order to analyse the drawings collected from children, an inductive approach to qualitative content analysis was taken, as this permits a qualitative exploration of what is drawn alongside a quantitative assessment of the frequency with which specific categories emerge. Content analysis also makes it possible to consider whether these patterns differ as a function of other factors such as age and sex, whilst preserving the integrity and validity of coded data [24]. The content of children's drawings (qualitative data) was identified through nodes, which were grouped according to dimensions and lines of argument. The content of the children's drawings (qualitative data) was identified through codes, which were grouped into dimensions and narrative lines. Initially, a category guide was developed based on the researchers' experience and scientific evidence generated by similar methodological strategies using drawings [25, 26]. Reflexivity was maintained by the research team throughout the research process, discussing and challenging assumptions established. Three researchers acted as analysts, coming together to examine cases in which identified elements were ambiguous or difficult to label. Ambiguity was resolved through consensus between all coders. Comments and notes made by researchers according to pre-established criteria were re-evaluated by a third researcher. The researchers made conscious efforts not to accept potentially common assumptions at face value.

## 4. Results

The study identified a series of meaningful patterns pertaining to perceptions of the nursing figure through children's drawings. A total of 105 drawings were analysed by the software CAQDAS Atlas-ti. Sociodemographic information pertaining to participants is presented in Supporting Information. Of the 105 drawings, 47 corresponded to females and 38 to males. 20 participants chose not to provide information on gender. With regard to timeline, 26 drawings made prior to the onset of the COVID-19 were analysed and compared with 79 drawings from after the pandemic. In terms of familiarity with the nursing role, 33 drawings were made by children characterised by the presence of a relative who was a nurse, compared with 48 drawings for which this was not the case (Table 1).

Analysis of drawings produced 162 nodes and 971 citations, which were grouped into the following nine thematic categories: gender, uniform, use of tools, functions performed, decorative elements, represented individuals, use of text, work setting and facial expression. Furthermore, a category emerged from the discourse that was related to stereotypes and sexualisation of the profession. In order to promote representativeness and prevent isolated elements from swaying outcomes, nodes with degrees of freedom (df) greater than one were selected, in other words, nodes whose rooting or citation frequency was greater than one.

With regard to gender, girls demonstrated a higher tendency towards representing nurses in a specific and detailed way in their drawings (42 instances in girls vs. 22 instances in boys). In contrast, boys presented more neutral representations, indicating a lesser focus on the specific aspects of the profession. In addition, it was observed that girls more frequently included female nursing figures in their drawings in comparison with boys (40 instances in girls vs. 17 instances in boys).

**Figure 1:**
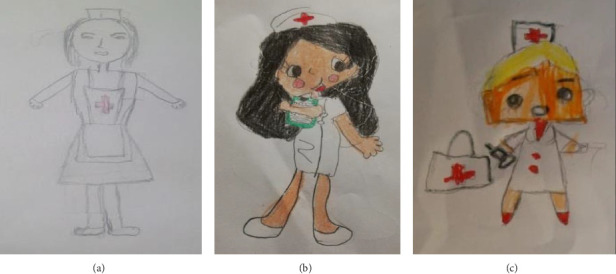
Illustrations that demonstrate the sexualisation and gender stereotypes associated with nursing. (a) Gender unknown, 10 years, prepandemic. (b) Gender unknown, nine years, postpandemic, with a relative in nursing. (c) 5-year-old girl, postpandemic, no relative in nursing.

As an emerging category, some drawings emerged depicting a stereotyped and sexualised nurse, wearing a uniform with a mob cap (57 instances), skirt (34 instances), heels (four instances) and, even, clearly notable makeup (10 instances). With regard to hair, girls more often drew nurses with loose hair (30 instances in girls vs. 20 instances in boys) (Figure 1).

**Figure 2:**
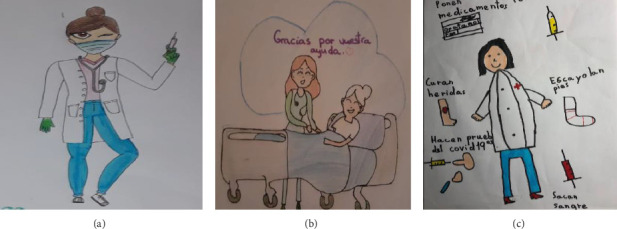
Illustrations that reflect the reality of nurses with regard to clothing and functions. (a) 11-year-old girl, postpandemic, no relative in nursing, (b) 12-year-old girl, postpandemic, with a relative in nursing, and (c) 7-year-old boy, postpandemic, no relative in nursing.

Nevertheless, it is important to highlight that, within the body of drawings analysed, representations are also found that do not adhere to the aforementioned stereotypes and are more closely aligned with reality. In these particular cases, social representations of nurses through drawings reflect clothing that is more recognisable as the professional uniform that characterises the profession (Figure 2).

When considering familiarity with the profession, it was observed that children who had a relative working in the profession drew more female nurses than those without relatives who worked in nursing (18 instances in those without nurse relatives vs. 35 instances in those with nurse relatives).

With regard to the time period (pre- or postpandemic), a greater representation of female nurses was found in drawings during the postpandemic period compared with prior to the pandemic (51 instances during the postpandemic vs. 21 instances prior to the pandemic). Likewise, it was notable that, during the postpandemic period, more nurses were drawn with their hair untied than during the prepandemic period (45 instances during the postpandemic period vs. 16 instances during the prepandemic period).

In terms of uniform, a very high occurrence was found of elements that are related to the history of the profession, which are no longer used, such as in the case of crucifixes (126 instances) and mob caps (57 instances), in addition to the sexualisation of the female nurse dressed in a skirt (34 instances). Nonetheless, characteristic elements were also found to be frequently depicted such as the nurse's uniform (45 instances), being represented through overalls (more characteristic of primary care or outpatient clinics) and the white or coloured scrubs (associated with the hospital setting). Girls showed a greater tendency than boys towards representing nurses in white scrubs (11 instances in girls vs. 6 instances in boys). Girls and boys coincided with regard to the frequency with which they included a crucifix in their drawings (11 instances each).

With regard to familiarity or family link with the profession, drawings made by children without relatives who worked in nursing more often depicted nurses with their hair tied back (9 instances in those without relatives in nursing vs. 0 instances in those with relatives in nursing). Furthermore, those with relatives working in nursing tended to represent nurses in coloured scrubs more often than those with relatives in nursing (13 instances in those without relatives in nursing vs. two instances in those with relatives in nursing). It was also observed that those with nursing relatives produced more depictions of nurses in plain clothing than those who did not have relatives working in nursing (8 instances in those with relatives in nursing vs. 1 instance in those without relatives in nursing).

Depictions of nurses wearing a scrub dress and with their feet up were more common in drawings made by those without relatives in nursing than those with relatives who worked in nursing (13 instances in those without relatives in nursing vs. 0 instances in those with relatives in nursing). Likewise, mob caps were more commonly represented in drawings made by those without relatives in nursing than those with relatives in nursing (28 instances in those without relatives in nursing vs. 15 instances than those with relatives in nursing).

Boys and girls included syringes in their drawings to the same extent (24 instances each). Nonetheless, girls were more inclined than boys to depict first aid kits (12 instances in girls vs. four instances in boys). In addition, both boys and girls drew hospital gurneys in similar proportions (21 instances overall). Finally, more girls included stethoscopes in their drawings than did boys (13 instances in girls vs. 7 instances in boys).

When examining familiarity with the profession, it was observed that children both with and without relatives who worked in nursing depicted syringes and first aid kits in similar proportions (24 instances depicting syringes and 15 instances depicting first aid kits). Nonetheless, more children without relatives in nursing drew hospital gurneys than those with relatives in nursing (14 instances in those without relatives in nursing vs. four instances in those with relatives in nursing), with more of the former group also drawing stethoscopes (15 instances in those without relatives in nursing vs. 5 instances in those with relatives in nursing).

Turning attention to the period (pre- or postpandemic), it was revealed that more paperwork, such as reports and graphs, was depicted during the postpandemic period than the period prior to the pandemic (22 instances postpandemic vs. 2 instances prepandemic), with the postpandemic period also being associated with a greater presence of syringes (22 instances postpandemic vs. 5 instances prepandemic), first aid kits (15 instances postpandemic vs. 2 instances prepandemic), hospital gurneys (17 instances postpandemic vs. 9 instances prepandemic) and stethoscopes (20 instances postpandemic vs. 2 instances prepandemic). Characteristic elements associated with COVID-19 also emerged such as drawings of viruses and superhero capes (Table S1).

The most commonly represented function was giving injections (29 instances overall), with this term being used to encapsulate the administration of both injections and vaccines. When analysing outcomes according to familiarity with the profession, it was observed that children without relatives in nursing placed greater emphasis on the role of giving injections than did those with relatives in nursing (17 instances in those without relatives in nursing vs. 6 instances in those with relatives in nursing). Likewise, during the postpandemic period, the function of giving injections was more frequently depicted than during the period prior to the pandemic (22 instances postpandemic vs. seven instances prepandemic). In the second place, the function of listening to a patient's heartbeat emerged (15 instances), followed by recording vital signs (9 instances) and healing (8 instances) (Table S2).

**Figure 3:**
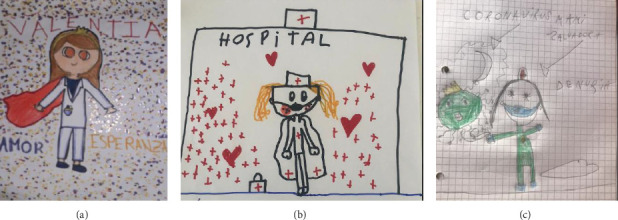
Illustrations reflecting the perceived image of nurses following the pandemic. (a) 9-year-old girl, postpandemic, no relative in nursing, (b) 7-year-old girl, postpandemic, with a relative in nursing, and (c) 6-year-old girl, postpandemic, with a relative in nursing.

Whilst the two most commonly depicted functions during the prepandemic period were giving injections (7 instances) and healing (3 instances), following the pandemic, new functions emerged in drawings such as saving (2 instances), which, as can be seen in Figure 3, is pictorially reinforced through the addition of elements such as a superhero cape.

With regard to associated decorative elements, girls tended to include more hearts in their drawings (12 instances in girls vs. 2 instances in boys). This frequent depiction of hearts in drawings made by girls may indicate an emotional or affective association with the nursing profession, suggesting that the girls have more positive and empathetic perceptions of nurses as carers and providers of emotional support.

Children both with and without relatives who worked in the profession included hearts in their drawings to a similar extent (6 instances in those with relatives in nursing vs. 8 times in those without relatives in the profession), suggesting that views on this element are shared by those with and without a familial link to nursing.

In the postpandemic period, an accentuated presence of hearts emerged in children's drawings (14 instances postpandemic), highlighting this element as being novel to this stage, together with other aspects such as flowers, viruses, rainbows and prohibition.

With regard to represented individuals, 31 of the 105 drawings presented a depiction of the nurse together with a patient, and in 10 drawings, more than one professional was depicted. Both boys and girls exhibited a similar tendency towards drawing hospitals and doors. Overall, hospitals were drawn on 16 occasions, whilst doors were drawn on 13 occasions.

In consideration of familiarity with the profession, children both with and without relatives who worked in the profession also depicted hospitals and doors to a similar extent, with hospitals being drawn on a total of 15 occasions and doors on a total of 13 occasions.

In the postpandemic period, greater representation of hospitals and doors was observed relative to in the prepandemic period (15 instances postpandemic vs. 3 instances prepandemic and 11 instances postpandemic vs. 4 instances prepandemic for hospitals and doors, respectively).

Finally, it was observed that boys more frequently depicted happy expressions in their drawings in comparison with girls (28 instances in boys vs. 23 instances in girls).

Turning attention to familiarity with the profession, children without relatives who worked in nursing more often depicted happy expressions than did those who had relatives in the profession (33 instances in those without relatives in nursing vs. 15 instances in those with relatives in nursing).

Finally, the situation brought about by the pandemic negatively influenced the frequency with which happiness was depicted in the expressions of nurses illustrated in drawings. Specifically, prior to the pandemic, 69.23% (18 instances) of drawings exhibited a happy facial expression, whilst after the pandemic, the proportion of drawings with happy expression decreased by 50% (46 instances).

## 5. Discussion

Perceptions of nursing figures held by children have started to be examined from the standpoint of children's representations in different studies [7, 27]. This has unveiled paradoxical aspects that englobe, on the one hand, ingrained stereotypes and, on the other, constantly evolving perceptions, with such outcomes also being reflected in the findings of the present study. One notable aspect in the drawings is the tendency to represent the nursing figure with elements such as skirts or high heels. However, some children show attire more in line with the typical uniform of nurses according to their speciality. This undermines contemporary efforts to challenge gender stereotypes and promote an up-to-date view of the nursing discipline [28]. Different studies conducted in Poland [29] and in Turkey [30] obtained similar results. This type of representation may reflect the influence of idealised or stereotypical images of the profession, which can lead to misconceptions, especially among children, who are most susceptible to such influences. These findings highlight the complexity of social and cultural influences on the construction of children's perceptions regarding this occupational field.

Differences in the representation of the nursing profession according to gender were highly evident in the present findings and reflect a finding of great interest. Whilst some studies suggest gender stereotypes do not exist [31], others show they persist [32]. Historically, the nursing profession has been overwhelmingly associated with the female gender. This perception has become deeply ingrained in the collective psyche [33] and explains why feminised representations of the profession abound in nursing. Indeed, this phenomenon is evidenced in the present study. Furthermore, this representation was reinforced in the sample analysed in the present study through the inclusion of symbolic elements, such as mob caps and crucifixes, which contributed to consolidating the traditional image of the nursing figure. The association between stereotyped images of nurses and traditional clothing impacts children's representations by perpetuating preconceived ideas about gender and societal roles. In this way, the need is highlighted to challenge and change these stereotypes in order to promote more inclusive and equative views of the profession of nursing.

Like other studies [34], the results obtained in this study show, in general terms, a positive perception of nurses. The results obtained in this study regarding the settings where children place nurses are also like those reported in other studies [30], such as attending to patients in the hospital room, in the treatment room, or in the emergency room. The children depicted nurses providing care and applying bandages. However, an interesting finding was that children, despite being too young to have seen nurses wearing caps, drew them with caps, even though nurses in Spain have not worn caps.

Another point of interest pertains to the pandemic's impact on children's perceptions of the nursing profession. The global health crisis has highlighted the crucial importance of the work performed by health professionals, including nurses. It is possible that recent events may have impacted the way in which children depict nurses in their drawings, with hospitals standing out as the main health institution in relation to which children visualise this figure. This aspect has already been highlighted by previously conducted studies [35] and can be seen through elements that support the recognition and valuing of their role in society, such as superhero capes, hearts and rainbows. All of these aspects emerged in the drawings examined in the present study.

### 5.1. Limitations

The volume of drawings made prior to the pandemic was notably less than the volume of drawings made in the stage following the pandemic, largely due to the interruption caused by COVID-19. This pause had a substantial impact on the production of drawings, limiting the number of drawings available for the prepandemic period. This uneven distribution of data between the two periods may affect the comparisons or conclusions drawn from the results.

Furthermore, the number of male participants was somewhat lower than of female participants. This gender imbalance in the sample should be considered when interpreting the findings as it may limit the generalisability of the results.

Another limitation encountered is the initial transformation from purposive sampling to snowball sampling. Whilst this approach allows access to participants with specific characteristics' relevant to the research, it presents certain limitations that must be considered.

## 6. Conclusions

Views held by children with regard to nursing figures are associated with previous experience and family links with this field of health professionals. To this end, the present study highlights the need to address perceptions from an early age, given that more accurate and realistic perceptions during childhood may lay the foundation for more positive and empathetic relationships with nurses in the future. In this sense, alongside other projective techniques, children's drawings provide effective research tools when working with minors. Indeed, this approach represents a highly expressive technique that provides a unique opportunity to explore and understand the views held by children of the nursing profession.

Findings of the present study revealed significant differences as a function of gender, familiarity with the profession and whether drawings were gathered prior to or following the pandemic. This suggests that it is important to consider these aspects as a means both of improving the relationship between children and nurses and, consequently, their experience of care, and of establishing a baseline of current perceptions of nurses, from which steps forward can be taken towards perceptions that are more in line with the reality of their labour.

Education and awareness raising are key tools for promoting a more accurate and respectful understanding of the nursing profession from an early age, challenging stereotypes and promoting respectful and collaborative relationships with nurses.

Research such as that presented here contributes towards extending the use of drawing as a useful tool for conducting research with children in the field of health. The need is emphasised to continue to explore and design educational strategies that promote a more accurate and respectful image of nursing from childhood.

## 7. Clinical Resources

This study provides an in-depth look at the tasks and responsibilities that children associate with nurses, which can help identify the aspects of the profession that are most attractive to children and the areas where education and public information about the profession need to be improved. In addition, understanding the social evolution of nursing from an early age can help professionals communicate with them more effectively.

## Figures and Tables

**Table 1:** Sociodemographic data of participants.

Characteristic	Frequency (*n*)	Percentage (%)
Age		
Preschool (< 6 years)	13	12.38
Primary (> 6 years)	54	51.43
Not reported	38	36.19
Sex		
Male	38	36.19
Female	47	44.76
Not reported	20	19.05
Timeline		
Prepandemic	26	24.76
Postpandemic	79	75.24
Familiarity with the nursing role		
Relative in nursing	33	31.43
No relative in nursing	48	45.71
Not reported	24	22.86

## Data Availability

The data that support the findings of this study are available from the corresponding author upon reasonable request.

## Supporting Information

The supporting data accompanying this manuscript include two additional tables. Table S1 provides the frequency with which tools linked to nursing identity are depicted. Table S2 presents the frequency with which the functions of nurses are depicted in drawings. This supporting information aims to provide further insight and context to support the findings presented in the main text.
