# Supplementary material for: Social Representation, Stereotypes and Social Identity Pertaining to Nursing Through Children's Drawings: A Qualitative Study
Source: Nurs Res Pract. 2024 Dec 31;2024:2417051. doi: 10.1155/nrp/2417051 (PMC11707062; doi:10.1155/nrp/2417051)
Supplement: Supporting Information — Additional supporting information can be found online in the Supporting Information section. [file 2417051.f1.docx]

|  | Girls | | Boys | | Relative in nursing | | No relative in nursing | | Pre-pandemic | | Post-pandemic | |
| --- | --- | --- | --- | --- | --- | --- | --- | --- | --- | --- | --- | --- |
|  | **n** | **%** | **n** | **%** | **n** | **%** | **n** | **%** | **n** | **%** | **n** | **%** |
| Syringe df=27 | 13 | 48.15% | 11 | 40.74% | 9 | 33.33% | 14 | 51.85% | 5 | 18.52% | 22 | 81.48% |
| Gurney df=26 | 11 | 42.31% | 10 | 38.46% | 4 | 15.38% | 14 | 53.85% | 9 | 34.62% | 17 | 65.38% |
| Stethoscope df=22 | 13 | 59.09% | 7 | 31.82% | 5 | 22.73% | 15 | 68.18% | 2 | 9.09% | 20 | 90.91% |
| First aid kit  df=17 | 12 | 70.59% | 4 | 23.53% | 9 | 52.94% | 6 | 35.30% | 2 | 11.76% | 15 | 88.24% |
| Reports df=11 | 5 | 45.45% | 3 | 27.27% | 2 | 18.18% | 7 | 63.64% | 2 | 18.18% | 9 | 81.82% |
| Pens df=9 | 4 | 44.44% | 3 | 33.33% | 4 | 44.44% | 3 | 33.33% | 4 | 44.44% | 5 | 55.56% |
| Table df=6 | 2 | 33.33% | 2 | 33.33% | 1 | 16.67% | 3 | 50% | 2 | 33.33% | 4 | 66.67% |
| Ambulance df=4 | 0 | 0% | 3 | 75% | 3 | 75% | 0 | 0% | 1 | 25% | 3 | 75% |
| Medicine df=4 | 0 | 0% | 3 | 75% | 0 | 0% | 3 | 75% | 1 | 25% | 3 | 75% |
| Pills df=4 | 2 | 50% | 1 | 25% | 2 | 50% | 1 | 25% | 1 | 25% | 3 | 75% |
| Vaccine  df=3 | 1 | 33.33% | 2 | 66.67% | 1 | 33.33% | 2 | 66.67% | 0 | 0% | 3 | 100% |
| Cart  df=3 | 1 | 33.33% | 1 | 33.33% | 0 | 0% | 2 | 66.67% | 1 | 33.33% | 2 | 66.67% |
| Otoscope  df=2 | 1 | 50% | 1 | 50% | 0 | 0% | 1 | 50% | 1 | 50% | 1 | 50% |
| Blood bag  df=2 | 1 | 50% | 1 | 50% | 1 | 50% | 1 | 50% | 0 | 0% | 2 | 100% |
| Plaster df=2 | 0 | 0% | 2 | 100% | 0 | 0% | 2 | 100% | 0 | 0% | 2 | 100% |

**Table S1. Frequency with which tools linked to nursing identity are depicted.**

**Table S2. Frequency with which the functions of nurses are depicted in drawings.**

|  | Girl | | Boy | | Relative in nursing | | No relative in nursing | | Pre-pandemic | | Post-pandemic | |
| --- | --- | --- | --- | --- | --- | --- | --- | --- | --- | --- | --- | --- |
|  | **n** | **%** | **n** | **%** | **n** | **%** | **n** | **%** | **n** | **%** | **n** | **%** |
| Injections df=29 | 12 | 41.38% | 11 | 37.93% | 6 | 20.69% | 17 | 58.62% | 7 | 24.14% | 22 | 75.86% |
| Auscultate df=15 | 8 | 53.33% | 6 | 40% | 3 | 20% | 11 | 73.33% | 1 | 6.67% | 14 | 93.33% |
| Record vital signs df=9 | 4 | 44.44% | 3 | 33.33% | 1 | 11.11% | 7 | 77.78% | 1 | 11.11% | 8 | 88.89% |
| Heal df=8 | 2 | 25% | 5 | 62.5% | 3 | 37.5% | 3 | 37.5% | 3 | 37.5% | 5 | 62.5% |
| Help  df=5 | 3 | 60% | 0 | 0% | 2 | 40% | 1 | 20% | 2 | 40% | 3 | 60% |
| Patient care df=5 | 1 | 20% | 3 | 60% | 1 | 20% | 3 | 60% | 1 | 20% | 4 | 80% |
| Administer medicine df=3 | 1 | 33.33% | 2 | 66.67% | 1 | 33.33% | 2 | 66.67% | 0 | 0% | 3 | 100% |
| Take blood df=2 | 1 | 50% | 1 | 50% | 1 | 50% | 1 | 50% | 0 | 0% | 2 | 100% |
| Save df=2 | 2 | 100% | 0 | 0% | 2 | 100% | 0 | 0% | 0 | 0% | 2 | 100% |
| Putting on a cast df=2 | 0 | 0% | 2 | 100% | 0 | 0% | 2 | 100% | 0 | 0% | 2 | 100% |
